# Sustainable Extraction of Bioactive Phenolics from Rose Hips for Functional Food Applications: Evaluation of Green Solvents and Extraction Techniques

**DOI:** 10.3390/foods14142448

**Published:** 2025-07-11

**Authors:** Hanna Kaczkowska, Marharyta Pestriakova, Jolanta Wółkiewicz, Aneta Krakowska-Sieprawska, Paweł Fijałkowski, Zbigniew Rafiński, Paweł Pomastowski, Justyna Walczak-Skierska, Katarzyna Rafińska

**Affiliations:** 1 Department of Environmental Chemistry and Bioanalytics, Faculty of Chemistry, Nicolaus Copernicus University, Gagarina 7 St., 87-100 Torun, Poland; 307435@stud.umk.pl (H.K.); 316186@stud.umk.pl (M.P.); 503571@doktorant.umk.pl (P.F.); katraf@umk.pl (K.R.); 2 Laboratory for Instrumental Analysis, Faculty of Chemistry, Nicolaus Copernicus University, Gagarina 7 St., 87-100 Torun, Poland; jola12@umk.pl (J.W.); payudo@chem.umk.pl (Z.R.); 3 Interdisciplinary Centre of Modern Technologies, Nicolaus Copernicus University, Wileńska 4 St., 87-100 Torun, Poland; aneta_krakowska@wp.pl (A.K.-S.); p.pomastowski@umk.pl (P.P.)

**Keywords:** green solvents, γ-valerolactone (GVL), Cyrene™, ethyl lactate, rose hips, phenolic, compounds, antioxidant activity, sustainable extraction, bioactive compounds

## Abstract

Growing interest in sustainable functional food ingredients has accelerated the search for green extraction methods for bioactive compounds. This study systematically evaluates the use of three emerging green solvents, namely γ-valerolactone (GVL), Cyrene™, and ethyl lactate (EL), as alternatives to conventional solvents for extracting phenolic antioxidants from rose hip (*Rosa canina* L.) fruit. Using maceration, ultrasound-assisted extraction (UAE), and microwave-assisted extraction (MAE), we compared extraction efficiency, total phenolic content, and antioxidant activity across various solvent systems and techniques. Our results demonstrate that MAE consistently provided the highest extraction yields and phenolic recovery, particularly when using ethanol or ethanol/green solvent mixtures. While pure green solvents showed lower extraction efficiency than ethanol, certain binary mixtures, especially GVL with ethanol, delivered promising results both in phenolic yield and antioxidant activity, without significant interference in standard assays. Additionally, while Cyrene™ consistently yielded low extraction efficiencies and low levels of phenolic compounds, its extracts were unique in exhibiting selectivity and stimulated fibroblast migration in vitro, suggesting additional functional benefits for health applications. Overall, our findings support the practical use of selected green solvents in sustainable extraction protocols for food, nutraceutical, and cosmetic industries.

## 1. Introduction

The beauty and fragrance of roses have been admired by humankind since ancient times, with the earliest written records appearing in ancient Chinese and Sanskrit texts [1]. Today, *Rosa canina*, commonly known as the wild rose, is widely regarded as the “queen of flowers” and is extensively used in the cosmetic, pharmaceutical, and food industries [2,3,4,5].

The increasing demand for functional foods enriched with natural antioxidants has prompted a search for novel, sustainable sources and extraction processes for bioactive compounds. Rose hips (*Rosa* spp.) are recognized as a particularly rich source of phenolic compounds, which contribute to their well-documented antioxidant, anti-inflammatory, and health-promoting properties. The integration of rose hip extracts into functional food products not only adds value in terms of nutritional benefits, but also aligns with consumer preferences for clean-label, plant-derived ingredients [5,6,7,8,9].

Traditionally, the recovery of phenolics from plant materials has relied on the use of organic solvents such as ethanol, methanol, water, hexane, acetone, chloroform, and ethyl acetate that have historically been used for the extraction of phenolic compounds from a variety of plant materials. However, due to their environmental impact and limitations in large-scale applications, these solvents are increasingly being phased out. As a result, there has been a growing demand for less-toxic, more-sustainable alternatives that also offer high extraction efficiency [10,11]. While these solvents are effective, their use poses environmental and health concerns due to toxicity, flammability, and challenges in waste management. In recent years, there has been a growing emphasis on adopting green chemistry principles in extraction processes, with a particular focus on environmentally benign, non-toxic, and biodegradable solvents collectively known as “green solvents.” Among these, bio-based options such as ethyl lactate, γ-valerolactone, and Cyrene™ have emerged as promising substitutes for traditional organic solvents, offering both improved safety profiles and reduced environmental impact [11,12,13,14,15].

Although their application to *Rosa canina* has not been previously reported, these solvents have demonstrated excellent performance in extracting polyphenols from related plant matrices. For instance, GVL has been used to extract antioxidant phenolics from kiwifruit waste [16], while ethyl lactate has been successfully applied to the recovery of flavonoids from *Cytisus scoparius* [17]. Cyrene™, derived from cellulose, has shown selective solubilization properties toward polar bioactives, although it requires careful handling due to its high viscosity and potential assay interference [16].

In addition to solvent selection, advances in extraction technology have enabled more efficient and selective recovery of target phytochemicals [10]. Modern techniques such as ultrasound-assisted extraction (UAE) and microwave-assisted extraction (MAE) not only improve extraction yields but also reduce energy consumption and processing times compared to traditional maceration. The combination of innovative extraction methods with green solvents represents a significant step toward the sustainable production of high-value bioactive ingredients from plant resources [18,19,20,21].

Despite these advancements, the suitability of green solvents and their compatibility with various extraction methods for the recovery of phenolic compounds from rose hips remain insufficiently explored. Moreover, the influence of solvent properties and extraction selectivity on the antioxidant activity and functional quality of the resulting extracts warrants thorough investigation. Therefore, the present study aims to systematically evaluate the efficiency of selected green solvents—Cyrene™, γ-valerolactone, and ethyl lactate—in combination with different extraction techniques for the recovery of bioactive phenolics from rose hips. This work addresses not only the extraction yields and antioxidant potential of the obtained extracts, but also provides insight into the sustainability and practical applicability of these approaches for future functional food formulations.

## 2. Materials and Methods

### 2.1. Plant Material

The plant material used was dried wild rose (*Rosa canina* L.) fruit from PPHU “AWB” Alina Becla Handzlówka, Poland, holding an organic production and trade certificate PL-EKO-01-5971, issued by EKOGWARANCJA PTRE sp. z o.o., and authorized by the Ministry of Agriculture, Poland. This confirms the organic origin of the fruits used in the study. The product consisted of 100% sliced and cleaned rose hip fruits, with no additives, preservatives, or sulfur treatment. Approximately 1 kg of dried material corresponds to ~7 kg of fresh rose hips (after deseeding). According to the manufacturer, the nutritional composition per 100 g of dried rose hip was as follows: energy 1077 kJ/260 kcal; fat 2.1 g (including 0.2 g saturated fatty acids); carbohydrates 82.1 g (including 19.1 g sugars); fiber 52.2 g; protein 4.4 g; salt 0.01 g. The dried plant material was ground into a fine powder using a laboratory mill (particle size < 1 mm) and stored in the dark until further analysis.

### 2.2. Extraction Methods

Maceration, ultrasound-assisted extraction (UAE), and microwave-assisted extraction (MAE) were performed under controlled laboratory conditions using standardized protocols. In this study, 96% ethanol (Honeywell, Seelze, Germany, puriss p.a.) and binary mixtures of green solvents were used as extraction media. The green solvents—Cyrene™ (Sigma Aldrich, Steinheim, German, BioRenewable, purity 99%), γ-valerolactone (Sigma Aldrich, Steinheim, German, BioRenewable, purity ≥ 99.0%), and ethyl lactate (Sigma Aldrich, Steinheim, German, purity ≥ 98.0%)—were combined with ethanol in weight ratios of 3:7 and 7:3. This approach enabled the evaluation of both pure solvents and mixed systems in terms of their efficiency for extracting bioactive phenolic compounds from rose hips.

Maceration was conducted by immersing 0.5 g of ground plant material in 10 mL of solvent in glass flasks. Samples were kept at 50 °C under mixing conditions for 24 h. The extracts were centrifuged at 14,000 rpm for 20 min and stored in a refrigerator until further analysis.

Ultrasound-assisted extraction (UAE) was carried out in an ultrasonic bath (Polsonic, Warsaw, Poland) operating at a frequency of 40 kHz. A total of 0.5 g of plant material was mixed with 10 mL of solvent in a glass beaker and sonicated two times for 30 min at 50 °C.

Microwave-assisted extraction (MAE) was performed using a Microwave Discover equipped with Synergy^TM^.exe.Ink software. Approximately 0.35 g of plant material was suspended in 7 mL of solvent and subjected to microwave irradiation at 600 W for 10 min. After extraction, the samples were allowed to cool, before being centrifuged and stored at 4 °C until further analysis.

### 2.3. Determination of Dry Matter Content (DM)

Extraction yield was determined gravimetrically based on the dry residue obtained from maceration, ultrasound-assisted extraction (UAE), and microwave-assisted extraction (MAE). A 1.0 mL aliquot of each extract was transferred into pre-weighed Eppendorf tubes and evaporated to constant weight at 90 °C for seven days. The procedure was performed in triplicate for each extraction method. The extraction yield was calculated according to the following equation:

$$Y_{extract}\left(\%\right)=\frac{m_{extract}}{m_{feed}}\times 100$$

where *m_extract_* is the dry mass of the extract (g) and *m_feed_* is the initial mass of the plant material used for extraction (g).

### 2.4. Total Phenolic Content (TPC)

Total phenolic content in the extracts was assessed using a modified colorimetric Folin–Ciocalteu assay, based on the procedure originally proposed by Singleton et al. [22]. Overall, 12 μL of extract was combined with 188 μL of deionized water and 12 μL of Folin–Ciocalteu reagent. The mixture was allowed to react in darkness for 8 min. Subsequently, 38 μL of a 20% sodium carbonate solution was added to initiate color development. After a 30 min incubation at 20 °C in the absence of light, absorbance was measured at 765 nm using a Varioskan™ LUX multimode plate reader (Thermo Fisher Scientific, Waltham, MA, USA). A blank containing all reagents except the sample was used for calibration. Results were calculated as gallic acid equivalents (GAE) and expressed in mg GAE per gram of dry extract.

### 2.5. DPPH Method

The antioxidant activity of the extracts was assessed using the DPPH (2,2-diphenyl-1-picrylhydrazyl) radical scavenging assay, with slight modifications based on the method reported by Espín et al. [23]. A 200 μL aliquot of 0.1 mM DPPH solution in ethanol (initial absorbance adjusted to 0.90 ± 0.02) was mixed with 50 μL of the extract in a 96-well microplate. The samples were incubated in the dark at room temperature for 30 min. Absorbance was recorded at 517 nm using a Varioskan™ LUX multimode microplate reader (Thermo Fisher Scientific, Waltham, MA, USA). All measurements were performed in triplicate. Results were expressed as micromoles of Trolox equivalents per gram of dry extract (μmol TE/g d.e.).

### 2.6. MALDI-TOF-MS Analysis

For MALDI-TOF-MS analysis, 0.5 μL of extracts obtained via maceration, ultrasound-assisted extraction (UAE), and microwave-assisted extraction (MAE) was deposited onto a ground steel MALDI target plate. Mass spectrometric measurements were performed using an UltrafleXtreme II MALDI-TOF/TOF mass spectrometer (Bruker Daltonics, Bremen, Germany) equipped with a smartbeam II Nd:YAG laser operating at 355 nm wavelength and 2 kHz repetition rate. Spectra were recorded in positive reflector mode with an acceleration voltage of 25 kV, covering a mass-to-charge (*m*/*z*) range of 100–4500 Da. Data acquisition and processing were conducted using flexControl and flexAnalysis version 3.4 software (Bruker Daltonics). Cluster analysis was further performed using ClinProTools 3.4 software (Bruker Daltonics).

### 2.7. Scanning Electron Microscopy

The morphology of rose hip materials subjected to different extraction techniques—namely maceration, ultrasound-assisted extraction (UAE), and microwave-assisted extraction (MAE)—was analyzed using scanning electron microscopy (SEM) with a Quanta 3D FEG instrument.

### 2.8. Cytotoxicity and Scratch Assay

The L929 cell line, from the European Collection of Authenticated Cell Cultures (ECACC), was used for all experiments. Cells were cultured in Dulbecco’s Modified Eagle Medium (DMEM) supplemented with 10% (*v*/*v*) fetal bovine serum, 2 mM glutamine, 100 U/mL penicillin, and 100 μg/mL streptomycin. Upon reaching approximately 80% confluence, the cells were passaged using 0.25% trypsin/EDTA. For viability assays, cells were seeded into 96-well plates at a density of 2 × 10^5^ cells/mL. After 24 h, the medium was replaced with fresh medium containing the tested extracts at a concentration of 0.5 mg/mL, and the cells were incubated for an additional 24 h. Next, 10% (*v*/*v*) MTT solution (5 mg/mL in PBS) was added to each well and incubated for 3 h. Following incubation, the medium was removed, and the formazan crystals were dissolved in DMSO with gentle shaking for 10 min. Absorbance was measured at 570 nm with background correction at 650 nm using a Multiskan microplate reader (ThermoFisher).

A wound healing assay was also performed to evaluate the effects of silver and zinc nanocomposites on cell migration and wound closure. L929 cells were seeded into 24-well plates at a density of 1 × 10^6^ cells per well and cultured for 24 h to achieve a confluent monolayer. Linear scratches were made using a sterile 100 μL pipette tip, followed by rinsing with PBS to remove detached cells. Images of the wounds were captured at 0 h, after which cultures were treated with tested extracts at concentration 0.5 mg/mL. Mobility of fibroblasts was monitored by acquiring images at 48 h post-treatment.

## 3. Result and Discussion

Research on the extraction of bioactive compounds from rose hips (*Rosa canina* L.) emphasizes the evaluation of various extraction techniques and environmentally friendly solvents, which is of critical importance to both the food industry and environmental sustainability. Among the investigated methods, ultrasound-assisted extraction (UAE) and microwave-assisted extraction (MAE) are recognized as modern, sustainable approaches that comply with the principles of green chemistry, and provide significant advantages for the recovery of natural bioactives. This study aims to assess the extraction efficiency of the selected green solvents—γ-valerolactone, Cyrene™, and ethyl lactate—using three different techniques, namely maceration, UAE, and MAE, along with a comparative analysis of their effectiveness in extracting phenolic compounds, carotenoids, and polyphenols.

### 3.1. Extraction Efficiency

**Figure 1:**
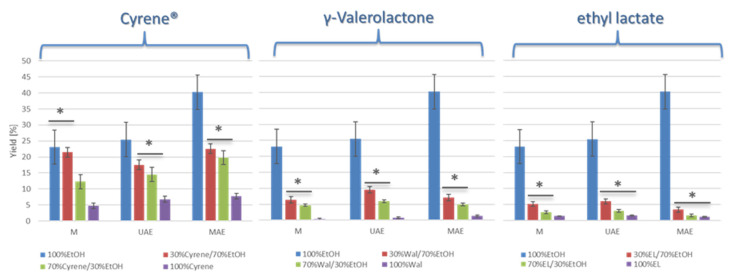
Extraction efficiency in extracts obtained by M, UAM, MAE depending on solvent and solvent mixture. Asterisks (*) denote pairwise comparisons that were not statistically different according to Tukey’s HSD test (*p* > 0.05). No marker indicates statistically significant differences (*p* ≤ 0.05).

The extraction efficiency of bioactive compounds from *Rosa canina* varied significantly depending on both the solvent system used and the extraction method applied. For all solvent systems and extraction methods, the use of 96% ethanol consistently resulted in the highest extraction yields, with values reaching approximately 40.24% *w*/*w* for MAE (Figure 1 and Table 1). Regardless of the solvent or technique, MAE (microwave-assisted extraction) outperformed both UAE and maceration, yielding significantly higher extract percentages. This confirms the effectiveness of MAE in enhancing mass transfer and cell wall disruption, thus facilitating more efficient recovery of soluble solids.

In contrast, the introduction of green solvents such as Cyrene™, γ-valerolactone, or ethyl lactate, either as pure solvents or in binary mixtures with ethanol, led to a marked decrease in extraction yields. For each solvent group, extraction efficiency declined as the proportion of the green solvent increased. Pure Cyrene™, γ-valerolactone, and ethyl lactate were notably less effective, resulting in minimal yields irrespective of the extraction method used. Binary mixtures with ethanol achieved slightly higher yields but remained substantially lower than those obtained with pure ethanol. The obtained results demonstrate that while green solvents are promising from a sustainability perspective, their extraction efficiency, particularly in pure form, remains lower than that of conventional ethanol.

### 3.2. Total Phenolic Content (TPC)

The most commonly used method for the determination of total phenolic content is the Folin–Ciocalteu assay, owing to its simplicity, sensitivity, and broad applicability across a wide range of sample types. This colorimetric method is based on the reduction of the Folin–Ciocalteu reagent by phenolic compounds under alkaline conditions. Upon reduction, a blue-colored complex is formed, the intensity of which can be quantitatively measured by spectrophotometry at wavelengths typically ranging from 760 to 765 nm.

The reaction is non-specific to individual phenolics but is responsive to the overall reducing capacity of the sample, making it suitable for the estimation of total phenolic content (TPC). Results are generally expressed in terms of gallic acid equivalents (GAE), using a calibration curve prepared with standard solutions of gallic acid. The Folin–Ciocalteu method is widely employed for the qualitative assessment of ethanol, methanol, acetone, and aqueous extracts, particularly in the analysis of plant-derived materials. To date, relatively few studies have focused on the determination of total phenolic content in extracts obtained using green solvents, such as deep eutectic solvents (DES), natural deep eutectic solvents (NADES), or bio-based solvents. These environmentally friendly alternatives to traditional organic solvents are gaining popularity due to their low toxicity and sustainability. However, their complex chemical composition and potential reducing capacity may interfere with the Folin–Ciocalteu assay, leading to an overestimation of phenolic content. Therefore, careful method optimization and appropriate controls are essential when applying this assay to extracts prepared with green solvents.

We evaluated the applicability of the Folin–Ciocalteu method for the qualitative analysis of extracts obtained using green solvents such as Cyrene™, γ-valerolactone, and ethyl lactate. To this end, a test was performed employing pure solvents and their binary mixtures with ethanol to assess their potential interference with the assay. This approach enabled us to determine the background absorbance and reducing properties of each solvent, which is critical for the accurate interpretation of results in phenolic content determination from extracts prepared with these environmentally friendly alternatives.

**Figure 2:**
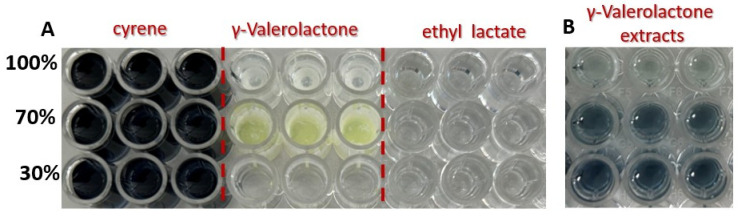
(**A**) Results of the Folin–Ciocalteu reaction for pure green solvents and their mixtures with ethanol: Cyrene™, γ-valerolactone, and ethyl lactate, and their mixtures with ethanol demonstrating their individual color responses and potential for assay interference. (**B**) Effect of the Folin–Ciocalteu reaction with extracts obtained with γ-valerolactone and its mixtures.

Both pure Cyrene™ and its binary mixtures with ethanol produced a very intense positive response in the Folin–Ciocalteu assay, indicating strong reducing properties (Figure 2). This pronounced color development suggests that Cyrene™ itself can significantly interfere with the determination of total phenolic content, potentially leading to substantial overestimation of phenolics in extracts prepared with this solvent.

The strong interference observed for Cyrene™ in the Folin–Ciocalteu assay is directly related to its unique molecular structure. Cyrene™ contains a bicyclic system with carbonyl and ether functionalities, which impart significant reducing power. The presence of the carbonyl group, in particular, can facilitate electron transfer to the Folin–Ciocalteu reagent, resulting in the pronounced color reaction observed. This structural feature makes Cyrene™ much more reactive in the assay compared to other green solvents lacking such reducing groups, highlighting the importance of considering solvent structure when interpreting results of phenolic content determinations. Silva et al. also observed this phenomenon, reporting that Cyrene™ and its mixtures were the only solvents exhibiting intrinsic antioxidant activity possibly due to the presence of an aromatic ring in its structure. As a consequence, extracts obtained with Cyrene™ could not be reliably quantified using standard assays such as TPC, FRAP, or ABTS, and this solvent was excluded from subsequent analytical procedures in their study [16].

In the case of γ-valerolactone, precipitation was observed upon addition of the reagents used for the determination of total phenolic content (Figure 2). This phenomenon may hinder accurate spectrophotometric measurement by causing turbidity and light scattering, thereby complicating the assessment of phenolic content in extracts prepared with γ-valerolactone. Therefore, caution should be exercised when interpreting results obtained using γ-valerolactone as an extraction solvent. The absence of precipitation in extract samples does not rule out the possibility of matrix-dependent interactions or hidden interferences, which may affect the accuracy and reliability of phenolic content determinations. Careful validation and appropriate controls are recommended when applying the Folin–Ciocalteu assay to samples prepared with γ-valerolactone.

It is only for ethyl lactate that we did not observe any adverse effects or interfering changes. This solvent did not produce significant background absorbance or precipitation in the presence of the Folin–Ciocalteu reagents, indicating its suitability for use in the determination of total phenolic content by this method.

The observations described above posed a significant challenge for further analyses. Consequently, for samples extracted with Cyrene™, we decided to evaporate the solvent and replace it with ethanol prior to performing the Folin–Ciocalteu assay. This approach aimed to eliminate the strong interference caused by Cyrene™ and ensure more accurate determination of the total phenolic content. However, it should be noted that Cyrene™ has a high boiling point, which requires evaporation of the solvent to be performed at higher temperatures. As a result, some heat-sensitive compounds present in the extracts may undergo partial degradation during this process, potentially affecting the final phenolic profile and total content measured.

**Figure 3:**
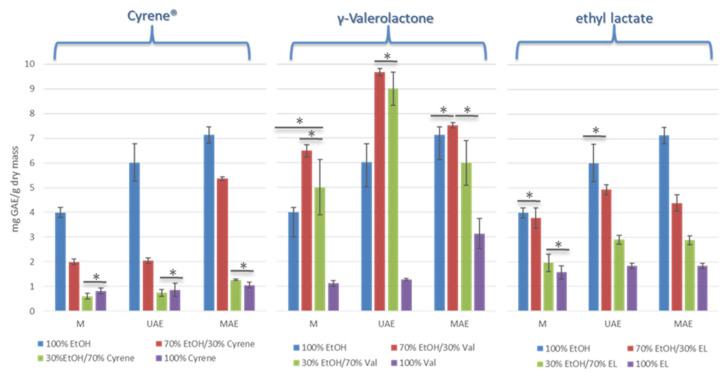
Total phenolic content of obtained rose hip fruit extracts using M, UAE, and MAE techniques. Asterisks (*) denote pairwise comparisons that were not statistically different according to Tukey’s HSD test (*p* > 0.05). No marker indicates statistically significant differences (*p* ≤ 0.05).

The results presented in Figure 3 demonstrate the substantial effect of both solvent composition and extraction technique on the determined total phenolic content. Across all extraction methods, the use of 96% ethanol yielded the highest total phenolic contents in rose hip extracts, with particularly notable results achieved using advanced techniques such as microwave-assisted extraction (MAE) and ultrasound-assisted extraction (UAE); in contrast, pure green solvents such as Cyrene™, γ-valerolactone, and ethyl lactate consistently resulted in significantly lower phenolic yields. Among all the green solvents tested, Cyrene™ was the least efficient in extracting phenolic compounds from rose hips, consistently yielding the lowest total phenolic content across all extraction techniques Similarly, the combination of Cyrene™ with ethanol in a 70/30% ratio was also notably ineffective, resulting in low phenolic yields comparable to those obtained with pure Cyrene™, regardless of the extraction technique used. We cannot exclude the fact that the high viscosity of Cyrene™ compared to ethanol can negatively impact extraction efficiency. Higher viscosity can hinder solvent penetration into plant matrices, limit mass transfer, and thereby reduce the extraction yield of phenolic compounds. Furthermore, Cyrene™ has limited miscibility with water, which may affect both the extraction process, as some phenolic compounds are more soluble in aqueous–organic mixtures than in pure organic solvents. However, it is worth noting that Milescu et al. observed substantially higher extraction efficiencies for Cyrene™ and its water mixtures up to three times higher than ethanol–water mixtures under similar conditions, and up to eleven times higher compared to older ethanol–water protocols, highlighting the fact that solvent composition and extraction parameters can strongly influence performance [24].

γ-Valerolactone stood out among the tested green solvents due to its noticeably higher efficiency in extracting phenolic compounds, especially when used in combination with ethanol (Table 2). The best results were achieved with the ethanol and γ-valerolactone mixture, particularly when ultrasound-assisted extraction (UAE) was applied; under these conditions, the total phenolic content was comparable to or even exceeded that obtained with pure ethanol. However, extraction efficiency with pure γ-valerolactone alone was considerably lower, indicating that combining this solvent with ethanol enables optimal exploitation of their extraction properties. The high effectiveness of γ-valerolactone mixtures likely results from its favorable influence on the solubility of certain phenolic groups and improved penetration into the plant matrix, which is especially evident when advanced extraction techniques are employed. These findings are consistent with those of Silva et al. [16], who demonstrated that among the alternative solvents studied, mixtures of γ-valerolactone with ethanol and/or water were the most effective for obtaining extracts with high levels of phenolic compounds and antioxidant activity, particularly at a γ-valerolactone:ethanol ratio of 7:3 (*w*/*w*). Among the techniques evaluated, microwave-assisted extraction (MAE) yielded the best outcomes, producing extracts with the highest total phenolic content and antioxidant activity at 50 °C.

Ethyl lactate demonstrated moderate efficiency as a green extraction solvent for phenolic compounds from rose hips. While the use of pure ethyl lactate generally resulted in lower total phenolic content compared to ethanol, combining ethyl lactate with ethanol in binary mixtures (especially at a 70% EtOH/30% EL ratio) improved extraction yields, particularly when modern techniques such as microwave-assisted extraction (MAE) or ultrasound-assisted extraction (UAE) were employed. Despite not matching the extraction efficiency of pure ethanol or the most effective γ-valerolactone mixtures, ethyl lactate-based systems exhibited good compatibility with the Folin–Ciocalteu assay and did not cause analytical interferences. This, along with its biodegradability and low toxicity, makes ethyl lactate a promising and safe green solvent for the extraction of bioactive phenolics in food and nutraceutical applications.

The obtained results highlight the importance of both the physicochemical properties of the extraction solvent—such as viscosity, solubility, and potential for assay interference—and the extraction method employed. While ethyl lactate and γ-valerolactone can be considered effective and compatible green solvents under certain conditions, the use of Cyrene™ requires special attention. Its high viscosity and strong reactivity in the Folin–Ciocalteu assay necessitate additional steps, such as solvent removal and replacement with ethanol, to achieve reliable and interpretable results for total phenolic content.

### 3.3. Antioxidant Activity (DPPH)

According to the literature, while the antioxidant activity of most solvents used for extraction is negligible, Cyrene™ and its mixtures have been reported to exhibit measurable antioxidant activity—potentially attributed to the presence of an aromatic ring in its structure. This unexpected activity led to challenges in quantifying antioxidant capacity using standard assays (such as TPC, FRAP, and ABTS), resulting in the exclusion of Cyrene™ from subsequent analytical steps in some studies [16]. In contrast, our results obtained with the DPPH assay showed that pure Cyrene™ did not cause any significant changes in absorbance, indicating the absence of inherent radical scavenging activity and suggesting that, at least for the DPPH method, Cyrene™ does not interfere with the detection of antioxidant compounds. This discrepancy may be explained by the different sensitivities or mechanisms underlying various antioxidant assays. The observed interference of Cyrene™ in the Folin–Ciocalteu assay, but not in the DPPH radical scavenging test, reflects the distinct chemical mechanisms underlying each method. The Folin–Ciocalteu assay is based on the reduction of a phosphomolybdate-phosphotungstate complex by electron-donating groups, such as hydroxyl or carbonyl moieties [25,26]. Cyrene™, containing a highly reactive carbonyl group, may act as a non-phenolic reductant in this system, leading to artificially elevated readings. In contrast, the DPPH assay involves hydrogen atom transfer (HAT) or single-electron transfer (SET) from antioxidant compounds to the DPPH radical [27]. Since Cyrene™ lacks sufficient hydrogen-donating capacity and is relatively inert toward stable free radicals, it does not interfere with this assay. This discrepancy underscores the importance of assay selection and solvent compatibility when evaluating antioxidant activity of extracts prepared with non-traditional solvents.

For the remaining tested green solvents—γ-valerolactone and ethyl lactate—no changes in absorbance were observed in the DPPH assay. This indicates that these solvents do not possess intrinsic radical scavenging activity and do not interfere with the detection of antioxidant compounds by this method. Consequently, γ-valerolactone and ethyl lactate can be considered compatible with the DPPH assay for the evaluation of antioxidant activity in plant extracts.

The results of the DPPH assay demonstrated that the antioxidant activity of the extracts varied depending on the extraction method and the solvent system employed. Ethanol-based extracts generally exhibited the highest antioxidant capacity compared to binary solvent mixtures.

**Figure 4:**
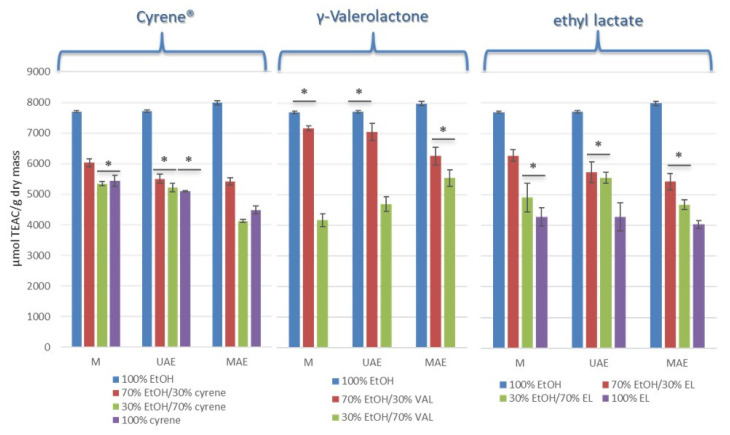
Antioxidant capacity of extracts obtained with different solvents and extraction techniques, expressed as μmol TEAC/g dry mass. Results are shown for extracts prepared using Cyrene™, γ-valerolactone, and ethyl lactate, in comparison to 100% ethanol, across three extraction methods: maceration (M), ultrasound-assisted extraction (UAE), and microwave-assisted extraction (MAE). Asterisks (*) denote pairwise comparisons that were not statistically different according to Tukey’s HSD test (*p* > 0.05). No marker indicates statistically significant differences (*p* ≤ 0.05).

The use of increasing concentrations of green solvents, particularly Cyrene™ and γ-valerolactone, led to a notable decrease in antioxidant capacity, likely reflecting differences in extraction efficiency and possible solvent-specific interferences. γ-valerolactone systems showed comparable performance to ethanol, especially when used in mixtures consisting of 70% ethanol and 30% γ-valerolactone, indicating its suitability as a green alternative for phenolic antioxidant extraction (Figure 4).

For both maceration (M) and ultrasound-assisted extraction (UAE), the highest antioxidant compound yield was achieved using γ-valerolactone, specifically with the 70% EtOH/30% GVL mixture, which recorded a value exceeding 7000 µmol TEA/g dry weight (Figure 3). In comparison, extracts obtained with pure ethanol under the same conditions exhibited even higher antioxidant activity, reaching approximately 8000 µmol TEA/g dry weight.

Although extracts obtained with mixtures of γ-valerolactone and ethanol demonstrated a higher total phenolic content (TPC), they exhibited comparatively lower antioxidant activity than extracts prepared with pure ethanol. This suggests that the phenolic compounds extracted in the presence of γ-valerolactone may possess lower antioxidant potential or that γ-valerolactone preferentially extracts phenolic species with weaker reducing properties. As a highly polar, aprotic solvent, GVL, particularly in combination with ethanol, may preferentially solubilize glycosylated flavonoids, phenolic acids, or polymeric phenolics, which are generally less redox-active than their aglycone counterparts. While such compounds still react with the Folin–Ciocalteu reagent due to their reducing capacity, they often show lower radical scavenging efficiency in assays like DPPH, which rely on direct electron or hydrogen transfer to a stable free radical. Indeed, it has been shown that glycosylation of flavonoids reduces the availability of free hydroxyl groups, thereby diminishing their antioxidant capacity, even though they remain detectable by colorimetric TPC assays [28]. Alternatively, the presence of γ-valerolactone may also influence the extract matrix in a way that affects the outcome of antioxidant assays. Most notably, extracts obtained using pure γ-valerolactone did not exhibit any measurable antioxidant activity in the DPPH assay. This unexpected result suggests that either the extracted compounds lacked significant radical scavenging capacity, or that γ-valerolactone itself interfered with the assay, masking the presence of active phenolic constituents. A similar effect was described by Braga et al. [29], who reported a lack of correlation between high TPC and antioxidant activity in microwave- and freeze-dried turmeric leaves, attributing it to structural differences and the degradation of phenolic subclasses during processing.

The observed results highlight the significant impact of extraction selectivity on the antioxidant properties of the obtained extracts. The choice of solvent and extraction method determines not only the total yield of phenolic compounds, but also their qualitative profile, which in turn affects the measured antioxidant activity. Selectivity in extraction means that certain solvents such as γ-valerolactone or its mixtures with ethanol may preferentially extract specific groups of phenolic compounds or other matrix constituents with varying antioxidant capacities. As demonstrated, a higher total phenolic content does not necessarily translate to higher antioxidant activity, emphasizing the importance of considering both the quantity and quality of extracted compounds. This underscores the necessity for a comprehensive approach to extract characterization, combining total phenolic content determination with targeted antioxidant assays and, where possible, the profiling of individual phenolic species.

### 3.4. Selectivity of Extraction Methods

It is worth noting that although the total phenolic content of many of the tested extracts was lower per gram of dry plant material compared to those obtained with 96% ethanol, the high extraction yield achieved with ethanol suggests that this solvent ensures low selectivity [30,31,32]. In other words, ethanol tends to extract not only phenolic compounds but also a wide range of other plant constituents, which may dilute the concentration of phenolics in the total extract. This highlights the need to balance extraction efficiency with selectivity, depending on the intended application of the extract and the desired enrichment in bioactive compounds.

Therefore, the addition to total extraction yield and phenolic content, selectivity is a critical parameter when evaluating the performance of green extraction systems, particularly when the goal is to maximize recovery of target bioactive compounds, such as phenolics, while minimizing the co-extraction of undesired constituents.

**Figure 5:**
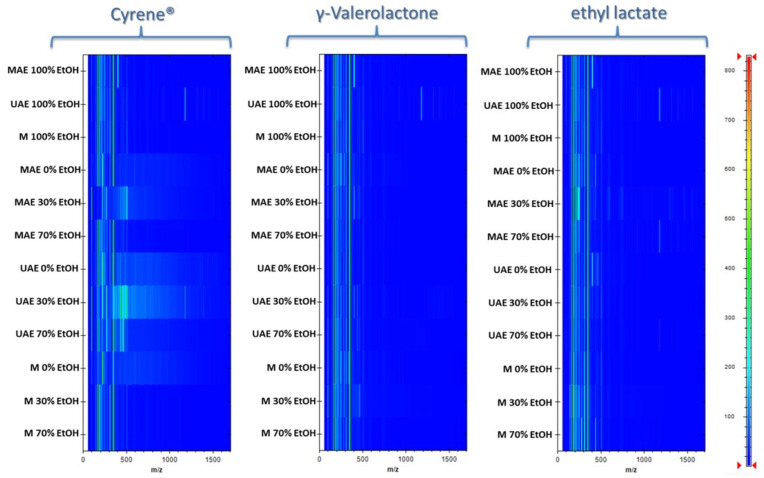
Gel-view MALDI-TOF MS spectra of rose hip extracts obtained using maceration (M), ultrasound-assisted extraction (UAE), and microwave-assisted extraction (MAE).

Gel-view MALDI-TOF MS analysis provided valuable insights into the molecular selectivity of the various extraction processes. Distinct molecular fingerprints were observed across different solvent systems, suggesting that each green solvent exhibited selective solubilization of specific classes of biomolecules (Figure 5). MALDI analysis revealed that, for extracts obtained using UAE with 96% ethanol, signals corresponding to ions with m/z values above 1000 were present, indicating the extraction of higher-molecular-weight compounds. In contrast, extracts prepared with solvent mixtures did not show signals in this higher mass range, suggesting that these solvents were less effective at extracting such macromolecular species. It is important to note that these high-molecular-weight interferents can be generally undesirable in phenolic extracts intended for functional food applications, as they may negatively affect the purity and stability of extracts. This highlights an additional advantage of green solvent mixtures, which offer improved selectivity and minimize the co-extraction of unwanted macromolecular components.

The molecular profile of extracts obtained using pure Cyrene™ or mixtures with a high Cyrene™ content was completely different from the molecular profile of extracts obtained with ethanol. The MALDI spectra for the Cyrene™-based systems show a distinct distribution and intensity of signals across the m/z range. These differences indicate that Cyrene™ selectively extracts a different set of compounds compared to ethanol, which is reflected in the altered molecular fingerprint of the resulting extracts.

For the other tested solvents, the distribution of signals was similar to that observed for ethanol. Both γ-valerolactone and ethyl lactate, as well as their mixtures with ethanol, produced molecular profiles closely resembling those of ethanol extracts, with a comparable range and intensity of ion signals across the m/z spectrum. This suggests that these green solvents, unlike Cyrene™, do not significantly alter the qualitative molecular composition of the extracts and are capable of extracting a similar set of compounds as ethanol.

The extraction efficiency and phenolic selectivity were found to be strongly influenced by the physicochemical properties of the solvents used, particularly their viscosity and polarity. As shown in Table 3, Cyrene™, despite its relatively high polarity, demonstrated lower extraction efficiency compared to ethanol. This can be attributed to its high viscosity, which impairs mass transfer and solvent penetration into plant matrices, especially under static extraction conditions such as maceration.

In contrast, γ-valerolactone (GVL), which has a lower viscosity and moderate polarity, enabled efficient extraction of mid-polar compounds, particularly when used in binary mixtures with ethanol. These mixtures appeared to offer a favorable balance between solvation power and diffusivity, enhancing the recovery of phenolic compounds without excessive co-extraction of interfering compounds.

Ethanol, as the least-viscous solvent used, ensured good mass transfer and high extraction yield, although its low selectivity may lead to the concurrent extraction of non-phenolic constituents. These observations highlight the need to consider both solvent polarity and viscosity in the design of green solvent systems for phenolic extraction and support the use of binary systems as a compromise between efficiency and selectivity.

**Figure 6:**
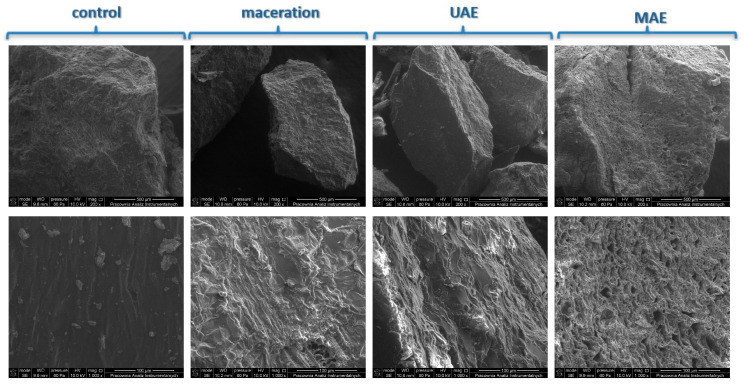
Representative SEM images of rose hip material after extraction by maceration, ultrasound-assisted extraction (UAE), and microwave-assisted extraction (MAE).

SEM images revealed distinct changes in the microstructure of plant tissues depending on the extraction method applied (Figure 6). Samples after maceration retained a relatively intact cellular structure, with visible and well-defined cell walls. In contrast, materials treated with UAE exhibited clear evidence of cell wall disruption and increased porosity, indicative of cavitation effects induced by ultrasonic waves. The most pronounced structural alterations were observed in samples subjected to MAE, where extensive cell wall rupture, fragmentation, and collapse were apparent. These observations confirm the enhanced effectiveness of UAE and MAE in facilitating the release of intracellular phenolic compounds by intensifying cell disruption compared to conventional maceration. Similar effects have been reported by Singh et al., who used SEM to analyze the microstructure of plant material after microwave-assisted extraction. They found that MAE led to a notably looser and more fragmented tissue structure compared to conventional extraction, which preserved intact, compact cell walls with few pores. According to Singh, the rapid internal heating and dielectric polarization generated by microwaves resulted in an explosive breakdown of cellular structures, significantly promoting the release of solutes—including polyphenolic compounds—into the solvent [32].

### 3.5. Environmental Impact of Tested Extraction Methods

A practical assessment of the extraction methods reveals not only differences in yield and selectivity, but also significant variations in energy consumption and operational costs. Conventional maceration conducted at 50 °C for 24 h was the most energy-intensive method, requiring approximately 2.4 kWh per batch. In addition, the long extraction time is correlated with increased labor and the potential risk of compound degradation over extended thermal exposure, making this technique less favorable for high-throughput or large-scale applications. Ultrasound-assisted extraction (UAE), carried out at 200 W for 60 min, consumed roughly 0.20 kWh per batch. Microwave-assisted extraction (MAE) was the most energy-efficient, with typical extractions performed at 600 W for 10 min, equating to just 0.10 kWh per batch. MAE not only achieved rapid extraction and higher yields for certain solvent systems, but also minimized thermal degradation and solvent loss due to its short processing time. Additionally, the relatively simple operation and reduced footprint of modern microwave reactors make MAE a highly attractive option for both laboratory and potential industrial scale-up. These observations are consistent with previous reports by Chemat et al. [34], who demonstrated that UAE significantly reduce energy input, solvent consumption, and CO_2_ emissions when compared to traditional methods such as maceration or Soxhlet extraction. For instance, UAE applied to oilseeds reduced energy demand by more than 90% and carbon emissions by over 95% relative to conventional protocols [35].

From a cost perspective, both UAE and MAE offer significant advantages over traditional maceration by lowering energy and labor expenses and increasing throughput. However, the initial investment in equipment for MAE and UAE is higher than for conventional heating setups. Over time, the operational savings from reduced energy usage and processing times can offset these costs, particularly in settings where process efficiency and sustainability are priorities.

### 3.6. Scratch Assay

Although plant extracts are recognized as valuable sources of bioactive compounds, their application in food, pharmaceutical, or biomedical fields requires careful safety assessment due to the possibility of adverse effects on eukaryotic cells. To assess the safety of the tested extracts and rule out potential cytotoxic effects, a cytotoxicity assay was performed using the mouse fibroblast cell line L929, in accordance with the EN ISO 10993-5 standard. At a concentration of 0.5 mg/mL, none of the tested extracts demonstrated any cytotoxicity toward the L929 mouse fibroblast cell line, as assessed by the MTT viability assay.

**Figure 7:**
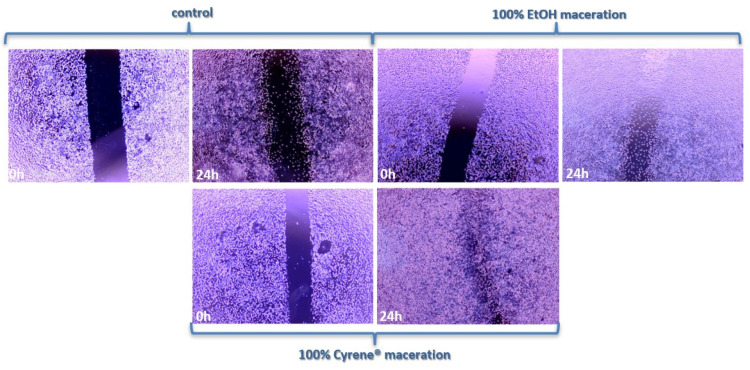
Scratch assay on L929 fibroblast cells.

The results of the wound-healing assay indicate that rose hip extracts can enhance fibroblast mobility in vitro (Figure 7). This effect suggests that bioactive compounds present in the extracts may stimulate fibroblast migration, a key process in tissue regeneration and repair. Although the molecular profile of the extract obtained with Cyrene™ differed markedly from that of the ethanol extract, the Cyrene™-derived extract exhibited a slightly stronger stimulatory effect on fibroblast migration compared to the ethanol extract. This suggests that, despite compositional differences, certain components selectively extracted by Cyrene™ may contribute more effectively to enhancing fibroblast mobility and, consequently, the regenerative potential of the extract.

From a food science perspective, the ability of rose hip extracts to support fibroblast mobility adds an important functional value, as it implies potential benefits for skin health and recovery when these extracts are incorporated into functional food products. Such properties are particularly relevant for consumers seeking dietary strategies to improve skin vitality, accelerate tissue regeneration, or support recovery from minor injuries and oxidative-stress-related damage.

## 4. Conclusions

This work demonstrates that green solvents such as γ-valerolactone and ethyl lactate, especially in combination with ethanol, can effectively replace traditional organic solvents for the extraction of phenolic antioxidants from rose hip fruits, contributing to more sustainable processing. Microwave-assisted extraction proved to be the most efficient technique, maximizing both yield and bioactive content. Although pure green solvents showed limited extraction capacities, mixtures with ethanol—particularly GVL/ethanol—offered a favorable balance between efficiency, selectivity, and environmental impact.

Based on the comparative performance of the tested solvent systems, the combination of ultrasound-assisted extraction (UAE) with 70:30 ethanol:GVL emerged as the most effective strategy for maximizing phenolic yield (9.67 ± 0.15 mgGAE/g dry mass) while maintaining antioxidant activity.

Cyrene™, while less effective for extracting antioxidant phenolics, enabled the selective isolation of compounds that enhanced fibroblast migration, suggesting potential value for use in functional foods or nutraceutical formulations aimed at supporting skin regeneration and tissue repair. Overall, the integration of green solvents with advanced extraction methods provides a promising route toward eco-friendly production of high-value plant extracts for functional foods and related sectors. These findings emphasize the need to align solvent choice with intended extract functionality and downstream application. Future work should focus on compound-specific profiling using HPLC-MS, solvent recyclability, and stability studies, to better understand extract composition, improve process sustainability, and support application in food and nutraceutical products.

## Figures and Tables

**Table 1:** Extraction yields [%] obtained using different solvent systems and extraction techniques (maceration, ultrasound-assisted extraction—UAE, microwave-assisted extraction—MAE). Data are expressed as mean ± standard deviation.

	Maceration Yield [%]	UAE Yield [%]	MAE Yield [%]
EtOH	23.11 ± 5.85	25.46 ± 5.03	40.24 ± 5.58
70% EtOH/30% Cyrene	21.48 ± 1.34	17.54 ± 1.40	22.56 ± 1.88
30% EtOH/70% Cyrene	12.32 ± 2.02	14.53 ± 1.87	19.80 ± 2.72
100% Cyrene	4.74 ± 0.96	7.77 ± 0.92	8.67 ± 0.86
70% EtOH/30% GVL	6.38 ± 0.99	9.58 ± 0.98	7.14 ± 1.30
30% EtOH/70% GVL	4.10 ± 0.35	5.97 ± 0.60	4.94 ± 0.50
100% GVL	0.40 ± 0.24	0.79 ± 0.15	1.38 ± 0.34
70% EtOH/30% EL	5.17 ± 0.75	5.99 ± 0.98	3.40 ± 0.67
30% EtOH/70% EL	2.60 ± 0.50	3.00 ± 0.77	1.57 ± 0.32
100% EL	1.40 ± 0.16	1.60 ± 0.19	1.19 ± 0.24

**Table 2:** Total phenolic content (TPC) of rose hip extracts obtained using different extraction techniques and solvent systems, expressed as mg gallic acid equivalents (GAE) per gram of dry weight (mg GAE/g DW). Results are presented as mean ± standard deviation.

	Maceration TPC (mg GAE/g Dry Mass)	UAE TPC (mg GAE/g Dry Mass)	MAE TPC (mg GAE/g Dry Mass)
EtOH	3.99 ± 0.20	6.02 ± 0.75	7.13 ± 0.33
70% EtOH/30% Cyrene	1.99 ± 0.12	2.06 ± 0.11	5.36 ± 0.08
30% EtOH/70% Cyrene	0.61 ± 0.12	0.74 ± 0.14	1.27 ± 0.03
100% Cyrene	0.83 ± 0.12	0.87 ± 0.26	1.06 ± 0.11
70% EtOH/30% GVL	6.49 ± 0.25	9.67 ± 0.15	7.46 ± 0.21
30% EtOH/70% GVL	5.01 ± 1.12	9.00 ± 0.67	5.97 ± 0.92
100% GVL	1.12 ± 0.12	1.27 ± 0.05	3.12 ± 0.76
70% EtOH/30% EL	3.79 ± 0.41	4.94 ± 0.17	4.39 ± 0.33
30% EtOH/70% EL	1.96 ± 0.35	2.90 ± 0.18	2.88 ± 0.18
100% EL	1.57 ± 0.28	1.84 ± 0.11	1.84 ± 0.11

**Table 3:** Physicochemical properties of selected green and conventional solvents relevant to phenolic extraction efficiency and selectivity. Data include viscosity, boiling point, and density at 25 °C. [33,34].

Solvent	Viscosity (cP 25°C)	Density (g/cm^3^)	Boiling Point (°C)	Character	Water Misciblity	Observed Yield
Ethanol	1.1	0.8	78.3	protic	complete	high
Cyrene^TM^	11.4 cP	1.25	227	aprotic	limited	moderate
γ-valerolactone	1.9 cP	1.05	207	aprotic	complete	low
Ethyl lactate	4.7 cP	1.03	154	protic	colplete	low

## Data Availability

The original contributions presented in the study are included in the article, further inquiries can be directed to the corresponding author.
